# Association of IS605 and *cag*-PAI of *Helicobacter pylori* Isolated from Patients with Gastrointestinal Diseases in Taiwan

**DOI:** 10.1155/2013/356217

**Published:** 2013-02-17

**Authors:** Chih-Ho Lai, Chin-Lin Perng, Keng-Hsin Lan, Hwai-Jeng Lin

**Affiliations:** ^1^Department of Microbiology, School of Medicine, China Medical University, Taichung 40402, Taiwan; ^2^Division of Gastroenterology, Department of Medicine, Taipei Veterans General Hospital, Taipei 11217, Taiwan; ^3^School of Medicine, National Yang-Ming University, Taipei 11221, Taiwan; ^4^Division of Gastroenterology and Hepatology, Department of Internal Medicine, Taipei Medical University Hospital, Taipei 11031, Taiwan; ^5^School of Medicine, Taipei Medical University, Taipei 11031, Taiwan

## Abstract

*Background*. The *cag* pathogenicity island (*cag*-PAI) is one of the most important virulent determinants of *Helicobacter pylori*. An insertion sequence (IS) element of *cag*-PAI (IS605) has been found to generate *H. pylori* strains with varying virulence. *Aim*. To evaluate the impact of IS605 and *cag*-PAI on *H. pylori* strains isolated from Taiwanese patients with severity of gastric diseases. *Methods*. *H. pylori* isolates were cultured from gastric biopsies from 99 patients with peptic ulcer, chronic gastritis, and gastric carcinoma. Six distinct, well-separated colonies were isolated from each patient and analyzed by genotyping. *Results*. *cagA*, *cagE*, *cagM*, *cagT*, *orf*10, and *orf*13 were found to be present in 90.0%–100.0% of the *H. pylori* isolates. A total deletion of *cagA*, *cagE*, *cagM*, *cagT*, *orf*10, and *orf*13 was found in 1 isolate (1.0%). The IS605 element was found to be positive in 15.2% of the isolates. The presence of IS605 was higher in *H. pylori* isolated from patients with gastric carcinoma (25.0%) than in patients with duodenal ulcer (6.5%) or chronic gastritis (6.3%) (*P* < 0.001). *Conclusions*. The majority of the patients examined had intact *cag*-PAI. IS605 was present in 15.2% and was higher in *H. pylori* isolated from patients with gastric carcinoma than in those with peptic ulcer.

## 1. Introduction

Infection with *Helicobacter pylori* is one of the most common bacterial infections in humans [1]. It has been closely linked with chronic gastritis, peptic ulcer, gastric carcinoma, and mucosa-associated lymphoid tissue (MALT) lymphoma [2]. The *cag *pathogenicity island (*cag*-PAI), a major determinant of *H. pylori* virulence, has been well studied [3, 4]. *cag*-PAI is an ~40-kb region in the *H. pylori* genome that contains a cluster of around 30 genes, including a type IV secretion system (T4SS) and the cytotoxin-associated gene A (CagA) [5]. Translocation of CagA into gastric epithelial cells requires delivery by T4SS and subsequent induction of cell signaling, which contributes to the development of pathogenesis in gastric mucosa [6].

**Figure 1:**
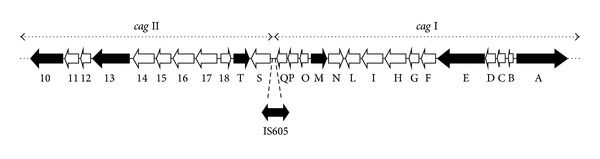
Structure of *H. pylori cag *pathogenicity island. The localizations of *cag* I, *cag* II, and IS605 are shown. The genes assessed in this study including *cagA*, *cagE*, *cagM*, *cagT*, *orf*10, and *orf*13 are indicated as close black.

An insertion sequence (IS) element, IS605, found in some strains disrupts an otherwise-uninterrupted *cag*-PAI unit, thereby splitting it into 2 regions (Figure 1). These regions, termed *cag *I and *cag *II, contain at least 14 and 16 open reading frames (*orf*s), respectively [7]. Insertion of IS605 between *cag* I and *cag *II generates *H. pylori* strains with different levels of virulence [8, 9]. Censini et al. reported that the *cagA* gene is closely linked to the intact *cag-*PAI [4]. However, the presence of the *cagA* gene does not guarantee the existence of an intact *cag*-PAI [10–12].

Diversity within *cag*-PAI is noted between the people belonging to the Eastern and Western hemispheres [13]. Only one-half to two-thirds of the isolates from the western world carry *cag*-PAI. In contrast, nearly all East Asian isolates carry *cag*-PAI [14, 15]. Therefore, *cagA* and other genes comprising the* cag*-PAI should be individually investigated. Moreover, *cag*-PAI appears to be disrupted in the majority of isolates globally [14]. Partial deletion of *cag*-PAI has been reported in 4%–88% isolates [12–14, 16]. However, the clinical relevance of strains with an intact *cag*-PAI remains controversial [17]. The aim of this study was to evaluate the impact of IS605 and *cag*-PAI on *H. pylori* strains isolated from Taiwanese patients with respect to disease severity.

## 2. Materials and Methods

### 2.1. Study Subjects

The study between January 2001 and September 2009 comprised patients who were examined for gastric ulcer, duodenal ulcer, chronic gastritis, or gastric carcinoma, as well as those who were clinically diagnosed with upper gastrointestinal problems. All had completed a self-administered questionnaire prior to being enrolled in the study. Patients were excluded from the investigation if they presented with any of the following: inability to give written informed consent, bleeding tendency (platelet count <50,000/mm^3^, prothrombin time 3 seconds more than controls, if on anticoagulants), or having taken H_2_-receptor antagonists or proton pump inhibitors within 2 weeks of enrollment. The study was approved by the Clinical Research Committee of the Veterans General Hospital, Taipei, Taiwan.

### 2.2. *H. pylori* Strains and Bacterial Culture

*H. pylori *isolates were cultured from gastric biopsy specimens and were identified by their positive reactivity for catalase, urease, and oxidase activities [18]. The isolates were cultured at 37°C on brain heart infusion (BHI) agar plates supplemented with 7% horse blood (containing nalidixic acid 10 *μ*g/mL; trimethoprim 5 *μ*g/mL, vancomycin 3 *μ*g/mL, and amphotericin 2 *μ*g/mL) under 12% CO_2_ with high humidity as in our previously reported study [19]. The rapid urease test was performed using an in-house urease test at room temperature for color change up to 24 hours. The test was defined as positive if the color changed from yellow to red [19, 20].

### 2.3. Polymerase Chain Reaction

After obtaining positive cultures from the biopsy specimens, 6 isolated colonies from a single culture plate were tested for genotypes with polymerase chain reaction (PCR). These colonies were homogenized in guanidinium isothiocyanate, using a sterile micropestle. DNA were extracted, washed, and eluted in 100 *μ*L of 10 mM Tris-Hcl (pH 8.3). Two microliters of the purified DNA was used for each PCR reaction. Eight primers were employed to assess the upstream and downstream of *cag*-PAI and IS605:* cagA*, *cagE*, *cagM, cagT*, *orf*10, *orf*13, and IS605 (*tnpA* and *tnpB*) (Table 1 and Figure 1). PCR was performed under the following conditions: 30 cycles at 94°C for 1 min, at 50.9°C–63°C for 2 min, and at 72°C for 1 min, and a final extension at 72°C for 5 min. PCR products were analyzed on 1.0% agarose gels. Mixed infection was defined if there was different *cagA*, *cagE*, *cagT*, *cagM*, and *orf*10, *orf*13 among the 6 isolates from one plate.

### 2.4. Statistical Analysis

Descriptive statistics are reported as the proportion for categorical variables with 95% confidence intervals (CI), and means ± standard deviation for continuous variables. The CIs for all proportions are calculated using the standard approximation of binomial. The Chi-square test with Fisher's exact test was used to compare the clinical variables and results. A *P* value of less than 0.01 was considered significant.

## 3. Results

Between January 2001 and September 2009, 449 patients (37 patients with chronic gastritis, 101 had duodenal ulcer, 140 with gastric ulcer, and 171 with gastric carcinoma) were enrolled in this study underwent endoscopic examination and *H. pylori* culture studies. Of the enrolled subjects, 234 patients were positive for *H. pylori* infection: 25 (68%) with chronic gastritis, 66 (65%) with duodenal ulcer, 77 (55%) with gastric ulcer, and 66 (39%) with gastric carcinoma. Among those patients, we randomly selected 99 subjects (16 patients with chronic gastritis, 31 with duodenal ulcer, 32 with gastric ulcer, and 20 with gastric carcinoma) for further analysis of their *H. pylori* isolates. Six distinct, well-separated colonies were obtained from each patient making a total of 594 isolates for *H. pylori* genotyping. In the 99 subjects, mixed infections of *H. pylori* strains were found in 11 (11.1%) patients.

Our data showed that* cagA*, *cagE*, *cagM*, *cagT*, *orf*10, and *orf*13 were present in between 90.0% and 100% of the isolates (Table 2). There were no differences in the *cag*-PAI status among isolates from patients with various clinical outcomes. Total deletion of *cagA*, *cagE*,* cagM*, *cagT*, *orf*10, and *orf*13 was found in 1 (1.0%) isolate from a patient with gastric carcinoma. There was only 1 isolate from patient with gastric ulcer had* cagA* deletion. Deletion of* cagM*,* cagT*, *orf*10, and *orf*13 was found in 2 (2.0%) isolates (1 isolate from patient with gastric carcinoma and 1 isolate with gastric ulcer), and *cagE* deletion was found in 4 (4.0%) isolates (each isolated from patient with gastric carcinoma, gastric ulcer, duodenal ulcer, or chronic gastritis, resp.). 

IS605 was found to be present in 15.2% (15/99) of the isolates in this study (Table 3). The presence of IS605 was significantly higher in the *H. pylori* strains isolated from patients with gastric carcinoma (5/20, 25.0%) than in patients with duodenal ulcer (2/31, 6.5%) or chronic gastritis (1/16, 6.3%) (*P* < 0.001). It was also higher in isolates from patients with gastric ulcer (7/32, 21.9%) compared to those with duodenal ulcer and chronic gastritis (*P* < 0.01).

## 4. Discussion

The total number of isolates used in this study was 594, and *cag*-PAI was found to be present in more than 96% of isolates, which is similar to that reported previously for eastern populations [12, 21–23]. Previous studies from the USA reported that* cagA* and* cagE* were detected in 66% and 62%* H. pylori* strains, respectively [24]. In other studies, *cagA*, *cagE*, *cagM*, and *cagT *were detected in 93%–100% of Korean and 80%–82% of the Colombian populations [13]. The presence of *cagA* and *cagE* in *H. pylori *isolated from Chinese, Indian, and Malay patients in Singapore ranged from 92.3% to 100% [12]. In southern Taiwan, *cagA*, *cagE*, and *cagT* were previously found to be present in 100% of the domestic strains [21]. Our present study of *H. pylori* isolates from patients in northern Taiwan yielded similar results, with *cagA*, *cagE*, *cagM*, and* cagT* being present in more than 96% of the isolates tested. 

Patients with intact *cag*-PAI are thought to be associated with more severe clinical outcomes [10, 25]. Maeda et al. from Japan reported that strains with partial deletions within the *cag*-PAI were only derived from patients with nonulcer dyspepsia, whereas strains with an intact *cag*-PAI originated only from patients with gastric carcinoma [11]. Jenks et al. reported that intact *cag*-PAI was found in 85% and 53% of duodenal ulcer and nonulcer dyspepsia isolates, respectively, [10]. They indicated that the clinical outcome of *H. pylori* infection was not reliably predicted by any gene of the *cag*-PAI [10]. However, since the deletion of *cag*-PAI has been observed in patients with peptic ulcer and nonulcer dyspepsia, the pathogenicity of *H. pylori* may therefore not be determined by *cag*-PAI, as suggested by Kawamura et al. [25].

IS605 is located between *cag *I and *cag *II in the prototype *H. pylori* and is thought to be closely related to *cag*-PAI [4]. However, a recent study has suggested that IS605 is not related to the *cag*-PAI status [26]. In a previous study involving the Taiwanese population, all isolates were reported as positive for the *cag*-PAI, but only 36% of these isolates carried an IS605 insertion [21]. The previous controversial study [21] may have used pooled cultures for the analysis, and the actual positive rate of IS605 may be lower because of the possibility of mixed infections.

Owen et al. reported that the distribution of genomic IS605 inserts varied widely with respect to patient disease severity [27]. In this study, IS605 was detected at an overall frequency of 15.2% of isolates and in 25.0% of isolates from patients with gastric carcinoma (Table 3). Thus the presence of IS605 was found to be higher in *H. pylori* from patients with gastric carcinoma. It was also higher in isolates from patients with gastric ulcer (21.9%) compared to those with duodenal ulcer (6.5%) and chronic gastritis (6.3%). These results were consistent with previous findings by Deguchi et al. who indicated that the presence of IS605 was more frequently associated with the* cag13* gene in gastric cancer patients [9]. Kersulyte et al. report that the IS605 element family has homology at the protein level with *orfB *(putative transposase gene) [28]; this gene is present in a *Salmonella *prophage and contributes to virulence during bacterial infection [29]. This evidence suggests that the presence of the IS605 element may have a possible modifying role with respect to strain pathogenicity [4]. However, the function of IS605 in relation to *H. pylori cag*-PAI and the severity of gastric diseases still remain unclear. Further studies are required to clarify the role of IS605 in the development of gastric carcinoma. 

In conclusion, a majority of patients infected with *H. pylori* contained intact *cag*-PAI. However, the presence of the *cagA* gene does not guarantee the presence of intact *cag*-PAI. The presence of IS605 is significantly higher in isolates from patients with gastric carcinoma compared to those with duodenal ulcer and chronic gastritis.

## Figures and Tables

**Table 1:** PCR primers used to detect *CagA, CagE, CagM*, *CagT, *orf*10, *orf*13, TnpA, *and* TnpB* in *H. pylori* isolates.

Genes	Primers	Nucleotide sequences (5′–3′)	Length of PCR products	Location in genome of reference^†^
*cagA *	cagA-F	GATAACAGGCAAGCTTTTGAGG	349	
	cagA-R	CTGCAAAAGATTGTTTGGCAGA		14994–15342
*cagE *	cagE-F	GTTACATCAAAAATAAAAGGAAGCG	735	
	cagE-R	CAATAATTTTGAAGAGTTTCAAAGC		12342–13076
*cagM *	cagM-F	ACAAATACAAAAAAGAAAAAGAGGC	587	
	cagM-R	ATTTTTCAACAAGTTAGAAAAAGCC		4815–9264
*cagT *	cagT-F	TCTAAAAAGATTACGCTCATAGGCG	490	
	cagT-R	CTTTGGCTTGCATGTTCAAGTTGCC		1182–1671
*orf*10	*orf*10-F	AATAGTGCTTTCTTTAGGATTAGCG	658	
	*orf*10-R	CCGATTTAATCCTTTCGCTTATGTG		8970–9627
*orf*13	*orf*13-F	CGTTCATGTTCCATACATCTTTGGC	617	
	*orf*13-R	GATTTATAGCGATCTAAGAAACCGC		704–1320
*tnpA *	tnpA-F	ATCAGTCCAAAAAGTTTTTTCTTTCC	338	
	tnpA-R	TAAGGGGGTATATTTCAACCAACCG		154–541
*tnpB *	tnpB-F	CGCTCTCCCTAAATTCAAAGAGGGC	578	
	tnpB-R	AGCTAGGGAAAAATCTGTCTATGCC		954–1531

^†^GenBank accession number. AF282853.1.

**Table 2:** Prevalence of *H. pylori *virulence factors among isolates from patients with gastric carcinoma, gastric ulcer, duodenal ulcer, and chronic gastritis.

Diagnosis^†^	GC	GU	DU	CG	Total
Number of isolates	20	32	31	16	99
*CagA *	20 (100.0%)	31 (96.8%)	31 (100.0%)	16 (100.0%)	98 (99.0%)
*CagE *	19 (95.0%)	31 (96.9%)	30 (96.8%)	15 (93.8%)	95 (96.0%)
*CagM *	19 (95.0%)	31 (96.8%)	31 (100.0%)	15 (93.8%)	96 (97.0%)
*CagT *	19 (95.0%)	31 (96.8%)	31 (100.0%)	16 (100.0%)	97 (98.0%)
*orf*10	18 (90.0%)	32 (100.0%)	31 (100.0%)	16 (100.0%)	97 (98.0%)
*orf*13	18 (90.0%)	32 (100.0%)	31 (100.0%)	16 (100.0%)	97 (98.0%)

^†^GC: gastric carcinoma; GU: gastric ulcer; DU: duodenal ulcer; CG: chronic gastritis.

**Table 3:** Prevalence of IS605 in *H. pylori* isolated from patients with gastric carcinoma, gastric ulcer, duodenal ulcer, and chronic gastritis.

Diagnosis^†^	Number (%) of IS605 positive isolates
GC (*n* = 20)	5 (25.0)*
GU (*n* = 32)	7 (21.9)^#^
DU (*n* = 31)	2 (6.5)
CG (*n* = 16)	1 (6.3)
Total (*n* = 99)	15 (15.2)

^†^GC: gastric carcinoma; GU: gastric ulcer; DU: duodenal ulcer; CG: chronic gastritis.

*P < 0.001, GC versus DU; GC versus CG.

^#^ *P* < 0.01, GU versus DU; GU versus CG.
